# Bis(3-aza­niumylpyridin-1-ium) hexa­chloridostannate(IV) dichloride

**DOI:** 10.1107/S1600536813006806

**Published:** 2013-03-16

**Authors:** Martin van Megen, Stephan Prömper, Guido J. Reiss

**Affiliations:** a Institut für Anorganische Chemie und Strukturchemie, Lehrstuhl II: Material- und Strukturforschung, Heinrich-Heine-Universität Düsseldorf, Universitätsstrasse 1, D-40225 Düsseldorf, Germany

## Abstract

The asymmetric unit of the title compound, (C_5_H_8_N_2_)_2_[SnCl_6_]Cl_2_, consists of one 3-aza­niumylpyridin-1-ium dication and one chloride ion in a general position and a hexa­chloridostannate(IV) dianion lying about a centre of inversion. The [SnCl_6_]^2−^ anion exhibits almost perfect octa­hedral geometry. The 3-aza­niumylpyridin-1-ium and chloride ions are connected *via* medium–strong charge-supported N—H⋯Cl hydrogen bonds, forming undulating layers in the (110) plane. The [SnCl_6_]^2−^ ions are located between these layers and occupy cavities formed by two facing layer puckers.

## Related literature

For related 3-aza­niumylpyridin-1-ium salts, see: Ali *et al.* (2008 ▶); Kapoor *et al.* (2012 ▶); Rao *et al.* (2011 ▶); Sarma *et al.* (2012 ▶); Willett *et al.* (1988 ▶). For related hexa­halogenido­metalate salts, see: Reiss (1998 ▶, 2002 ▶); Reiss & Helmbrecht (2012 ▶). For spectroscopy of hexa­chloridostannate(IV) salts, see: Brown *et al.* (1970 ▶); Ouasri *et al.* (2001 ▶). For graph-set theory and its applications, see: Bernstein *et al.* (1995 ▶); Etter *et al.* (1990 ▶).
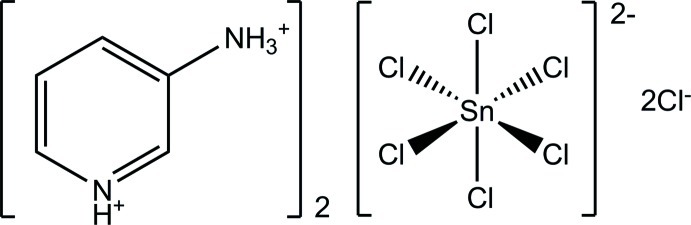


## Experimental

### 

#### Crystal data

(C_5_H_8_N_2_)_2_[SnCl_6_]Cl_2_
*M* *_r_* = 594.56
Orthorhombic, 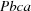

*a* = 11.9379 (3) Å
*b* = 10.3704 (3) Å
*c* = 16.7018 (5) Å
*V* = 2067.70 (10) Å^3^
*Z* = 4
Mo *K*α radiation
μ = 2.27 mm^−1^
*T* = 290 K
0.14 × 0.12 × 0.06 mm

#### Data collection

Oxford Diffraction Xcalibur Eos diffractometer
Absorption correction: multi-scan (*CrysAlis PRO*; Oxford Diffraction, 2009 ▶) *T*_min_ = 0.853, *T*_max_ = 1.000
30476 measured reflections
2364 independent reflections
1875 reflections with *I* > 2σ(*I*)
*R*_int_ = 0.038

#### Refinement

*R*[*F* ^2^ > 2σ(*F* ^2^)] = 0.020
*wR*(*F* ^2^) = 0.044
*S* = 1.06
2364 reflections
138 parameters
All H-atom parameters refined
Δρ_max_ = 0.26 e Å^−3^
Δρ_min_ = −0.22 e Å^−3^

### 

Data collection: *CrysAlis PRO* (Oxford Diffraction, 2009 ▶); cell refinement: *CrysAlis PRO*; data reduction: *CrysAlis PRO*; program(s) used to solve structure: *SHELXS97* (Sheldrick, 2008 ▶); program(s) used to refine structure: *SHELXL97* (Sheldrick, 2008 ▶); molecular graphics: *DIAMOND* (Brandenburg, 2012 ▶); software used to prepare material for publication: *publCIF* (Westrip, 2010 ▶).

## Supplementary Material

Crystal structure: contains datablock(s) I, global. DOI: 10.1107/S1600536813006806/pk2471sup1.cif

Structure factors: contains datablock(s) I. DOI: 10.1107/S1600536813006806/pk2471Isup2.hkl

Additional supplementary materials:  crystallographic information; 3D view; checkCIF report

## Figures and Tables

**Table 1:** Hydrogen-bond geometry (Å, °)

*D*—H⋯*A*	*D*—H	H⋯*A*	*D*⋯*A*	*D*—H⋯*A*
N1—H11⋯Cl1	0.94 (2)	2.23 (3)	3.135 (2)	161 (2)
N1—H12⋯Cl2^i^	0.91 (3)	2.48 (3)	3.343 (2)	160 (2)
N1—H13⋯Cl1^ii^	0.92 (3)	2.19 (3)	3.104 (2)	176 (2)
N2—H2⋯Cl1^iii^	0.86 (2)	2.21 (2)	3.055 (2)	167 (2)

Symmetry codes: (i) 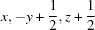; (ii) 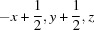; (iii) 
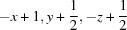
.

**Fig. 1.**
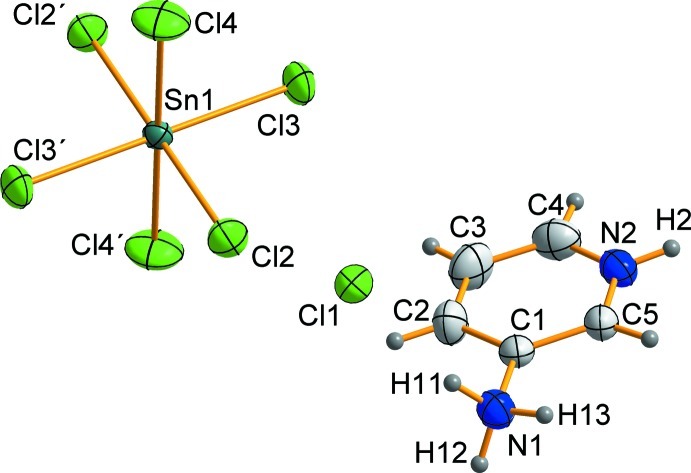
The asymmetric unit of the title compound plus the symmetry-related chlorido ligands completing the hexachloridostannate(IV) dianion (displacement ellipsoids are drawn at the 50% probability level; hydrogen atoms are drawn as spheres with arbitrary radii; symmetry code: -x, -y, -z).

**Fig. 2.**
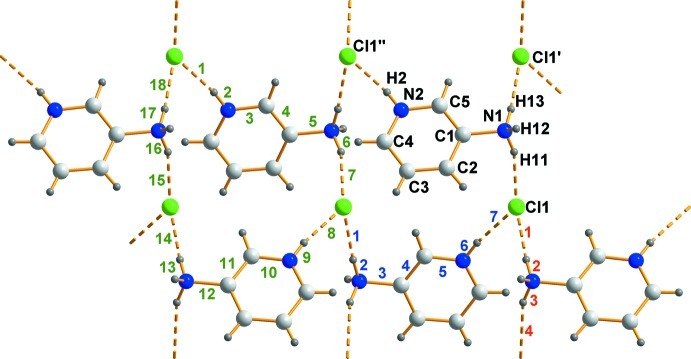
View on a hydrogen bonded layer of 3-azaniumylpyridin-1-ium dications and chloride anions in the [110] plane (graph-set descriptors R63(18), C21(4) and C21(7) are indicated with green, red and blue numbers; symmetry code: ' 0.5 - x, 0.5 + y, z, '' 1 - x, 0.5 + y, 0.5 - z).

**Fig. 3.**
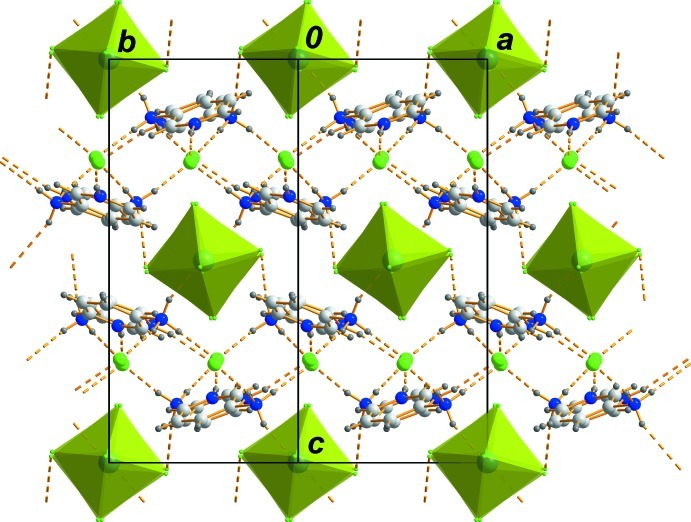
View along [110] on the title structure. Showing the hydrogen bonded layers consisting of 3-azaniumylpyridin-1-ium and chloride ions (parallel to ab at c = 1/4, 3/4) with the [SnCl6]2- dianions (parallel to ab at c = 0, 1/2, 1) located in cavities between them.

## supplementary crystallographic information

### Comment

Only a limited number of 3-azaniumylpyridin-1-ium containing compounds has structurally been characterized so far. The majority of these are salts consisting of halogenidometalate anions such as [BiCl_6_]^3-^ (Rao *et al.*, 2011), [CuCl_4_]^2-^ (Willett *et al.*, 1988), [CuBr_4_]^2-^ (Willett *et al.*, 1988) and [HgBr_4_]^2-^ (Ali *et al.*, 2008). Furthermore, the dinitrate (Kapoor *et al.*, 2012) and some complex crown ether compounds (Sarma *et al.*, 2012) have been reported. This study on (C_5_H_8_N_2_)_2_[SnCl_6_]Cl_2_ is part of our long standing interest on the principles of arrangement of simple hexahalogenidometalate salts (*dip*H = diisopropylaminium): (*dip*H)_2_[SiF_6_] (Reiss, 1998); (*dip*H)_2_[IrCl_6_] (Reiss, 2002); (*dip*H)_2_[SnCl_6_] (Reiss & Helmbrecht, 2012).

The asymmetric unit of the title compound, (C_5_H_8_N_2_)_2_[SnCl_6_]Cl_2_, consists of one 3-azaniumylpyridin-1-ium dication and one chloride ion in general positions and the hexachloridostannate(IV) dianion lying on a centre of inversion (Fig. 1). The C–N and C–C bond lengths and angles of the cation are within the expected ranges. The [SnCl_6_]^2-^ dianion exhibits a nearly perfect octahedral coordination sphere with Sn–Cl bond lengths ranging from 2.4162 (5) to 2.4242 (5) Å and bond angles between 88.03 (2) and 91.97 (2)°. The 3-azaniumylpyridin-1-ium dications and chloride ions are connected by medium strong, charge supported hydrogen bonds (Table 1) forming layers in the [110] plane. The characteristic hydrogen bonding motif is a 18-membered, wavy ring, which is classified as a third level graph-set *R*_6_^3^(18) (Etter *et al.*, 1990; Fig. 2). In addition to that, the second level graph-set descriptors C_2_^1^(4) and C_2_^1^(7) represent the chains, bridging the dications and chloride ions along [010] and [100], respectively. Thereby, the NH_3_^+^ group as well as the NH^+^ group of the 3-azaniumylpyridin-1-ium dication acts as a hydrogen bond donor. D···A distances for the NH_3_^+^ group range from 3.104 (2) to 3.343 (2) Å and for the NH^+^ group a D···A distance of 3.055 (2) Å is found. The [SnCl_6_]^2-^ dianions are located in cavities between the layers, connected to the 3-azaniumylpyridin-1-ium dications by weak hydrogen bonds (Table 1) between two of their chlorido ligands and neighbouring NH_3_^+^groups (Fig. 3). The Raman-active bands (ν_1_, ν_2_, ν_4_ and ν_5_) of the [SnCl_6_]^2-^ dianion appear in the Raman spectrum of the title compound (C_5_H_8_N_2_)_2_[SnCl_6_]Cl_2_ (Brown *et al.*, 1970; Ouasri *et al.*, 2001).

### Experimental

The title compound, (C_5_H_8_N_2_)_2_[SnCl_6_]Cl_2_, was prepared by dissolving 0.47 g (5.0 mmol) 3-aminopyridine and 0.65 g (2.5 mmol) tin(IV) chloride in 10 ml of concentrated (37%) hydrochloric acid. Within two to three days under ambient conditions colourless, rod-shaped crystals were obtained by slow evaporation of the solvent. The Raman spectrum was measured using a Bruker MULTIRAM spectrometer (Nd:YAG-Laser at 1064 nm; RT-InGaAs-detector); 4000–70 cm^-1^: 3082(w), 3049(w), 2954(vw), 1646(w), 1625(w), 1502(w), 1231(w), 1188(w), 1046(*m*), 1030(*m*), 815(w), 627(w), 537(w), 324(*vs*; ν_1_, Sn–Cl), 241 (m, br; ν_2_, Sn–Cl), 172 (s; ν_4_, Sn–Cl), 158 (s; ν_5_, Sn–Cl), 98 (m; most likely a lattice mode). - IR spectroscopic data were recorded on a Digilab FT3400 spectrometer using a MIRacle ATR unit (Pike Technologies); 4000–560 cm^-1^: 3458(w), 3364(w), 3245(w), 3187(w), 3080(*s*), 3066(*s*), 3008(*m*), 2952(*m*), 2880(*m*), 2773(*vs*), 2739(*vs*), 2687(*s*), 2604(*s*), 2542(*vs*), 1892(w, br), 1644(w), 1624(w), 1556(*s*), 1499(*s*), 1472(w), 1380(*m*), 1318(*m*), 1131(*m*), 1123(*m*), 1092(w), 1009(w), 998(w), 941(w), 890(w), 798(*m*), 673(*m*), 625(w). - Elemental analyses (C, H, N) were performed with a HEKA-Tech *Euro EA3000* instrument; SnCl_8_N_4_C_10_H_16_ (594.60): calcd. C 20.20, H 2.71, N 9.42; found C 20.38, H 2.54, N 9.34.

### Refinement

All hydrogen atoms were identified in difference syntheses and refined freely with individual *U*_iso_(H) values.

### Crystal data

**Table:**

(C_5_H_8_N_2_)_2_[SnCl_6_]Cl_2_	*F*(000) = 1160
*M**_r_* = 594.56	*D*_x_ = 1.910 Mg m^−^^3^
Orthorhombic, *P**b**c**a*	Mo *K*α radiation, λ = 0.71073 Å
Hall symbol: -P 2ac 2ab	Cell parameters from 11374 reflections
*a* = 11.9379 (3) Å	θ = 3.6–33.9°
*b* = 10.3704 (3) Å	µ = 2.27 mm^−^^1^
*c* = 16.7018 (5) Å	*T* = 290 K
*V* = 2067.70 (10) Å^3^	Block, colourless
*Z* = 4	0.14 × 0.12 × 0.06 mm

### Data collection

**Table:**

Oxford Diffraction Xcalibur Eos diffractometer	2364 independent reflections
Radiation source: fine-focus sealed tube	1875 reflections with *I* > 2σ(*I*)
Graphite monochromator	*R*_int_ = 0.038
Detector resolution: 16.2711 pixels mm^-1^	θ_max_ = 27.5°, θ_min_ = 3.6°
ω scans	*h* = −15→15
Absorption correction: multi-scan (*CrysAlis PRO*; Oxford Diffraction, 2009)	*k* = −13→13
*T*_min_ = 0.853, *T*_max_ = 1.000	*l* = −21→21
30476 measured reflections	

### Refinement

**Table:**

Refinement on *F*^2^	Primary atom site location: structure-invariant direct methods
Least-squares matrix: full	Secondary atom site location: difference Fourier map
*R*[*F*^2^ > 2σ(*F*^2^)] = 0.020	Hydrogen site location: difference Fourier map
*wR*(*F*^2^) = 0.044	All H-atom parameters refined
*S* = 1.06	*w* = 1/[σ^2^(*F*_o_^2^) + (0.0162*P*)^2^ + 0.6462*P*] where *P* = (*F*_o_^2^ + 2*F*_c_^2^)/3
2364 reflections	(Δ/σ)_max_ < 0.001
138 parameters	Δρ_max_ = 0.26 e Å^−^^3^
0 restraints	Δρ_min_ = −0.22 e Å^−^^3^

### Special details

**Table:**

Experimental. CrysAlisPro, Oxford Diffraction Ltd., Version 1.171.34.44 Empirical absorption correction using spherical harmonics, implemented in SCALE3 ABSPACK scaling algorithm.
Geometry. All e.s.d.'s (except the e.s.d. in the dihedral angle between two l.s. planes) are estimated using the full covariance matrix. The cell e.s.d.'s are taken into account individually in the estimation of e.s.d.'s in distances, angles and torsion angles; correlations between e.s.d.'s in cell parameters are only used when they are defined by crystal symmetry. An approximate (isotropic) treatment of cell e.s.d.'s is used for estimating e.s.d.'s involving l.s. planes.
Refinement. Refinement of *F*^2^ against ALL reflections. The weighted *R*-factor *wR* and goodness of fit *S* are based on *F*^2^, conventional *R*-factors *R* are based on *F*, with *F* set to zero for negative *F*^2^. The threshold expression of *F*^2^ > σ(*F*^2^) is used only for calculating *R*-factors(gt) *etc*. and is not relevant to the choice of reflections for refinement. *R*-factors based on *F*^2^ are statistically about twice as large as those based on *F*, and *R*- factors based on ALL data will be even larger.

### Fractional atomic coordinates and isotropic or equivalent isotropic displacement parameters (Å^2^)

**Table:**

	*x*	*y*	*z*	*U*_iso_*/*U*_eq_	
N1	0.28997 (16)	0.5670 (2)	0.35522 (13)	0.0399 (4)	
H11	0.262 (2)	0.496 (2)	0.3270 (14)	0.059 (8)*	
H12	0.257 (2)	0.574 (2)	0.4040 (16)	0.076 (9)*	
H13	0.273 (2)	0.639 (2)	0.3256 (14)	0.060 (8)*	
C1	0.41111 (17)	0.55437 (19)	0.36189 (11)	0.0308 (4)	
C2	0.4574 (2)	0.4437 (2)	0.39366 (14)	0.0433 (5)	
H2A	0.4103 (19)	0.381 (2)	0.4093 (13)	0.048 (7)*	
C3	0.5719 (2)	0.4332 (2)	0.39775 (15)	0.0511 (6)	
H3A	0.603 (2)	0.364 (2)	0.4159 (14)	0.061 (8)*	
C4	0.6373 (2)	0.5311 (3)	0.37014 (14)	0.0472 (6)	
H4A	0.712 (2)	0.529 (2)	0.3692 (14)	0.060 (8)*	
N2	0.58917 (16)	0.63570 (19)	0.33931 (10)	0.0395 (4)	
H2	0.630 (2)	0.695 (2)	0.3187 (14)	0.061 (8)*	
C5	0.47825 (17)	0.65048 (19)	0.33392 (12)	0.0335 (5)	
H5A	0.4526 (16)	0.7277 (19)	0.3111 (11)	0.033 (5)*	
Cl1	0.25614 (5)	0.31396 (5)	0.25548 (3)	0.04263 (13)	
Sn1	0.0000	0.0000	0.0000	0.02235 (6)	
Cl2	0.14393 (4)	−0.16245 (5)	0.01538 (3)	0.04262 (13)	
Cl3	−0.14685 (4)	−0.15423 (5)	0.02721 (3)	0.04332 (13)	
Cl4	0.00932 (5)	0.04893 (7)	0.14177 (3)	0.05529 (16)	

### Atomic displacement parameters (Å^2^)

**Table:**

	*U*^11^	*U*^22^	*U*^33^	*U*^12^	*U*^13^	*U*^23^
N1	0.0356 (11)	0.0370 (11)	0.0470 (11)	−0.0016 (9)	0.0044 (9)	0.0035 (10)
C1	0.0342 (11)	0.0297 (10)	0.0285 (9)	−0.0009 (9)	0.0042 (8)	−0.0031 (8)
C2	0.0491 (14)	0.0340 (12)	0.0469 (13)	−0.0021 (11)	0.0031 (11)	0.0082 (11)
C3	0.0556 (17)	0.0417 (14)	0.0559 (15)	0.0141 (13)	−0.0052 (12)	0.0085 (12)
C4	0.0357 (13)	0.0564 (15)	0.0493 (13)	0.0017 (12)	−0.0005 (11)	−0.0078 (12)
N2	0.0384 (11)	0.0414 (11)	0.0388 (10)	−0.0095 (9)	0.0066 (8)	−0.0026 (8)
C5	0.0391 (14)	0.0288 (10)	0.0327 (10)	−0.0016 (9)	0.0045 (8)	0.0004 (8)
Cl1	0.0474 (3)	0.0329 (2)	0.0476 (3)	0.0018 (2)	0.0113 (2)	−0.0002 (2)
Sn1	0.01879 (9)	0.02282 (9)	0.02543 (9)	−0.00037 (7)	0.00169 (7)	−0.00180 (6)
Cl2	0.0341 (3)	0.0371 (3)	0.0566 (3)	0.0133 (2)	0.0059 (2)	0.0094 (2)
Cl3	0.0334 (3)	0.0338 (3)	0.0627 (3)	−0.0120 (2)	0.0084 (2)	0.0002 (2)
Cl4	0.0572 (4)	0.0802 (4)	0.0285 (3)	0.0001 (3)	−0.0011 (3)	−0.0142 (3)

### Geometric parameters (Å, º)

**Table:**

N1—C1	1.456 (3)	C4—H4A	0.90 (3)
N1—H11	0.94 (2)	N2—C5	1.336 (3)
N1—H12	0.91 (3)	N2—H2	0.86 (2)
N1—H13	0.92 (3)	C5—H5A	0.938 (19)
C1—C5	1.362 (3)	Sn1—Cl3^i^	2.4162 (5)
C1—C2	1.380 (3)	Sn1—Cl3	2.4162 (5)
C2—C3	1.373 (4)	Sn1—Cl2^i^	2.4200 (5)
C2—H2A	0.90 (2)	Sn1—Cl2	2.4200 (5)
C3—C4	1.361 (4)	Sn1—Cl4	2.4242 (5)
C3—H3A	0.86 (2)	Sn1—Cl4^i^	2.4242 (5)
C4—N2	1.331 (3)		
			
C1—N1—H11	108.4 (16)	C5—N2—H2	117.0 (17)
C1—N1—H12	111.5 (17)	N2—C5—C1	118.4 (2)
H11—N1—H12	111 (2)	N2—C5—H5A	116.7 (12)
C1—N1—H13	109.8 (15)	C1—C5—H5A	124.9 (13)
H11—N1—H13	107 (2)	Cl3^i^—Sn1—Cl3	180.00 (3)
H12—N1—H13	109 (2)	Cl3^i^—Sn1—Cl2^i^	91.968 (19)
C5—C1—C2	120.3 (2)	Cl3—Sn1—Cl2^i^	88.032 (19)
C5—C1—N1	119.49 (19)	Cl3^i^—Sn1—Cl2	88.032 (19)
C2—C1—N1	120.1 (2)	Cl3—Sn1—Cl2	91.968 (19)
C3—C2—C1	119.0 (2)	Cl2^i^—Sn1—Cl2	180.00 (3)
C3—C2—H2A	123.4 (15)	Cl3^i^—Sn1—Cl4	90.68 (2)
C1—C2—H2A	117.7 (15)	Cl3—Sn1—Cl4	89.32 (2)
C4—C3—C2	119.7 (2)	Cl2^i^—Sn1—Cl4	89.46 (2)
C4—C3—H3A	119.5 (17)	Cl2—Sn1—Cl4	90.54 (2)
C2—C3—H3A	120.8 (17)	Cl3^i^—Sn1—Cl4^i^	89.32 (2)
N2—C4—C3	119.4 (2)	Cl3—Sn1—Cl4^i^	90.68 (2)
N2—C4—H4A	116.1 (16)	Cl2^i^—Sn1—Cl4^i^	90.54 (2)
C3—C4—H4A	124.5 (16)	Cl2—Sn1—Cl4^i^	89.46 (2)
C4—N2—C5	123.2 (2)	Cl4—Sn1—Cl4^i^	180.00 (5)
C4—N2—H2	119.7 (17)		

Symmetry code: (i) −*x*, −*y*, −*z*.

### Hydrogen-bond geometry (Å, º)

**Table:**

*D*—H···*A*	*D*—H	H···*A*	*D*···*A*	*D*—H···*A*
N1—H11···Cl1	0.94 (2)	2.23 (3)	3.135 (2)	161 (2)
N1—H12···Cl2^ii^	0.91 (3)	2.48 (3)	3.343 (2)	160 (2)
N1—H13···Cl1^iii^	0.92 (3)	2.19 (3)	3.104 (2)	176 (2)
N2—H2···Cl1^iv^	0.86 (2)	2.21 (2)	3.055 (2)	167 (2)

Symmetry codes: (ii) *x*, −*y*+1/2, *z*+1/2; (iii) −*x*+1/2, *y*+1/2, *z*; (iv) −*x*+1, *y*+1/2, −*z*+1/2.
